# Antibodies and vaccines against Middle East respiratory syndrome coronavirus

**DOI:** 10.1080/22221751.2019.1624482

**Published:** 2019-06-06

**Authors:** Jiuyang Xu, Wenxu Jia, Pengfei Wang, Senyan Zhang, Xuanling Shi, Xinquan Wang, Linqi Zhang

**Affiliations:** a Comprehensive AIDS Research Center, Collaborative Innovation Center for Diagnosis and Treatment of Infectious Diseases, Beijing Advanced Innovation Center for Structural Biology, Department of Basic Medical Sciences, Tsinghua University School of Medicine, Beijing, People’s Republic of China; b Ministry of Education Key Laboratory of Protein Science, Beijing Advanced Innovation Center for Structural Biology, Collaborative Innovation Center for Biotherapy, Tsinghua University School of Life Sciences, Beijing, People’s Republic of China

**Keywords:** Coronavirus, MERS-CoV, spike glycoprotein, monoclonal antibody, vaccine

## Abstract

The Middle East respiratory syndrome coronavirus (MERS-CoV) has spread through 27 countries and infected more than 2,200 people since its first outbreak in Saudi Arabia in 2012. The high fatality rate (35.4%) of this novel coronavirus and its persistent wide spread infectiousness in animal reservoirs have generated tremendous global public health concern. However, no licensed therapeutic agents or vaccines against MERS-CoV are currently available and only a limited few have entered clinical trials. Among all the potential targets of MERS-CoV, the spike glycoprotein (S) has been the most well-studied due to its critical role in mediating viral entry and in inducing a protective antibody response in infected individuals. The most notable studies include the recent discoveries of monoclonal antibodies and development of candidate vaccines against the S glycoprotein. Structural characterization of MERS-CoV S protein bound with these monoclonal antibodies has provided insights into the mechanisms of humoral immune responses against MERS-CoV infection. The current review aims to highlight these developments and discuss possible hurdles and strategies to translate these discoveries into ultimate medical interventions against MERS-CoV infection.

## GRAPHICAL ABSTRACT

**Figure:**
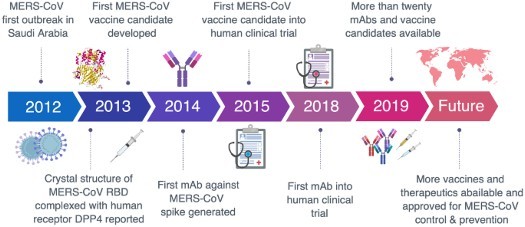

## 1. Introduction

The rapid emergence and dissemination of infectious diseases has taken a heavy toll on humans since the beginning of the twenty-first century. One of the most well-known examples was the outbreak of severe acute respiratory syndrome (SARS) in the winter of 2002 and 2003, caused by a novel coronavirus (SARS-CoV) [1,2]. In distinct contrast to the mild human coronaviruses HCoV-229E [3], HCoV-OC43 [4], HCoV-NL63 [5], and HCoV-HKU1 [6], infection with SARS-CoV frequently resulted in severe symptoms including fever, dry cough, shortness of breath and pneumonia. Transmission of SARS-CoV was primarily from person to person and most cases occurred in health care settings lacking adequate infection control precautions [2]. The SARS outbreak had severe consequences in 29 countries and regions, infecting 8096 people worldwide with a fatality rate of approximately 10% [7]. There are still no vaccines or therapeutics specific to SARS-CoV available 16 years after the SARS outbreak. It is not hard to imagine how catastrophic it would be if SARS-CoV were to hit the human community again.

While SARS-CoV remains a mystery and a loose cannon, another novel coronavirus emerged in Saudi Arabia in 2012, later known as the Middle East respiratory syndrome coronavirus (MERS-CoV) [8]. The fatality rate of MERS-CoV infection is approximately 35.4%, and new cases as well as associated deaths continue to arise to date [9]. Despite that most cases have been attributed to human-to-human transmission, MERS-CoV does not appear to transmit efficiently among humans unless there is close contact. The exact source of MERS-CoV and its routes of transmission to humans still remain uncertain. Dromedary camels are believed to be the animal reservoir for MERS-CoV because isolates from camels are almost identical to those from human, and that many domestic camels are seropositive for MERS-CoV (reviewed in [10,11]). Furthermore, current evidence strongly suggests that bats are the original source for MERS-CoV, as many coronaviruses phylogenetically related to MERS-CoV originate in bats, including BatCoV-HKU4, BatCoV-HKU5 and other MERS-related coronaviruses [12–15]. The BatCoV-HKU4 was also shown to be able to engage the cellular receptor of MERS-CoV, adding evidence to the bat origin theory [16]. However, there has not yet been direct evidence for isolating MERS-CoV from bats (reviewed in [10,11,17]).

Great efforts have been made to develop preventive and therapeutic interventions against MERS-CoV infection. In particular, monoclonal antibodies and vaccines targeting the Spike glycoprotein are major areas of focus due to its critical role in mediating viral entry, and its potential in inducing protective antibody responses in infected individuals. So far, more than twenty monoclonal antibodies with nanomolar neutralizing activities have been reported and many vaccine candidates are underway in preclinical and clinical studies. In this review, we aim to capture the current advances and discuss possible strategies to translate these discoveries into an ultimate medical intervention against MERS-CoV infection.

## 2. Structure and function of MERS-CoV spike glycoprotein

**Figure 1.**
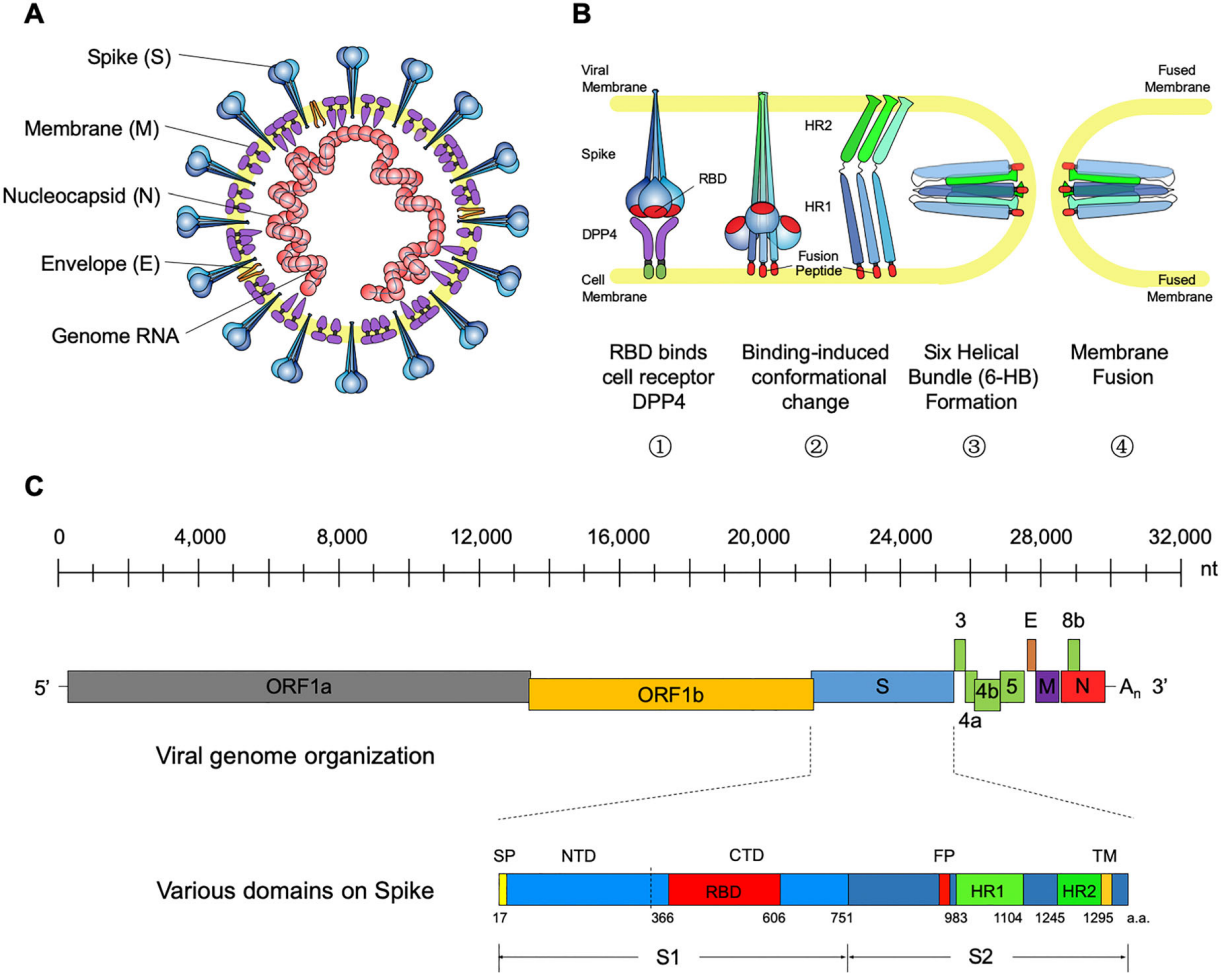
General introduction to MERS-CoV: model structure, life cycle and genomic composition. (A) Cartoon model structure of MERS-CoV. (B) Membrane fusion mechanism for MERS-CoV spike glycoprotein. Binding between RBD and the cell receptor (DPP4) triggers the conformational change of S glycoprotein to form a pre-hairpin intermediate of S2, in which the hydrophobic HR1 is exposed and the fusion peptide inserts into the target cell membrane. This transient S2 intermediate then refolds with HR2 into a stabilized trimer of hairpins, also called six-helix bundle structure (6-HB), bringing the target cell membrane into close proximity of the viral envelope and resulting in the completion of the fusion process. (C) Genomic composition of MERS-CoV. Each coloured box (length in scale) represents one open reading frame in the genomic RNA. The schematic for spike glycoprotein was also shown with labelled domain names and residue numbers. ORF (open reading frame), DPP4 (dipeptidyl peptidase 4), RBD (receptor-binding domain), NTD (N-terminal domain), CTD (C-terminal domain), FP (fusion peptide), and HR1-2 (heptad repeats 1-2).

MERS-CoV belongs to the genus *betacoronavirus* of the *coronaviridae* family [18]. It is an enveloped, single-stranded, positive-sense RNA virus with a helical capsid structure (Figure 1(A)). The genome of MERS-CoV is around 30 kb (30,119nt) long and encodes 4 structural proteins (Spike, Envelope, Membrane, and Nucleocapsid) and 16 nonstructural proteins (Figure 1(C)) [13]. Like other coronaviruses, the MERS-CoV uses its spike (S) glycoprotein to interact with cellular receptors and enter into the target cell [19–22]. As a unique structural component of the virion membrane, the S glycoprotein assembles into trimers and forms large protruding spikes on the surface of the virion [20]. The S glycoprotein is a typical type I membrane glycoprotein consisting of a globular S1 domain at the N-terminal, followed by a membrane-proximal S2 domain and a transmembrane (TM) domain [21]. The S1 domain mediates viral attachment and contains the RBD (receptor binding domain), which determines the host range and cellular tropism for MERS-CoV [23–25]. Similar to other coronaviruses, the S2 domain of MERS-CoV, mediating membrane fusion, contains the hydrophobic fusion peptide (FP) at the N-terminus as well as two heptad repeats designated as HR1 and HR2 (Figure 1(C)) [26]. Through co-purification with the MERS-CoV S1 domain, Raj and colleagues identified that dipeptidyl peptidase 4 (DPP4, also known as CD26) functions as a cellular receptor for MERS-CoV [27].

The MERS-CoV virion enters the host airway cells in the respiratory tract through fusion with either the plasma or endosomal membrane [19]. Binding between RBD and the cell receptor triggers a cascade of conformational changes that lead to the formation of a pre-hairpin intermediate of S2, in which the hydrophobic HR1 is exposed and allows the fusion peptide to insert into the target cell membrane. This transient S2 intermediate then refolds with HR2 into a stabilized trimer of hairpins, also called six-helix bundle structure (6-HB), which brings the target cell membrane into close proximity of the viral envelope, resulting in the completion of the fusion process and initiation of the virus life cycle [21] (Figure 1(B)). Structure-based design of various peptides able to block the formation of 6-HB have demonstrated potent inhibition on MERS-CoV replication and spike-mediated cell–cell fusion, showing great promise for further development into effective viral fusion inhibitors for treating MERS-CoV infection [26,28–30]. Among them, the peptide EK1 is effective to multiple human coronaviruses apart from MERS-CoV and therefore serves as a potential pan-coronavirus fusion inhibitor [30].

**Figure 2.**
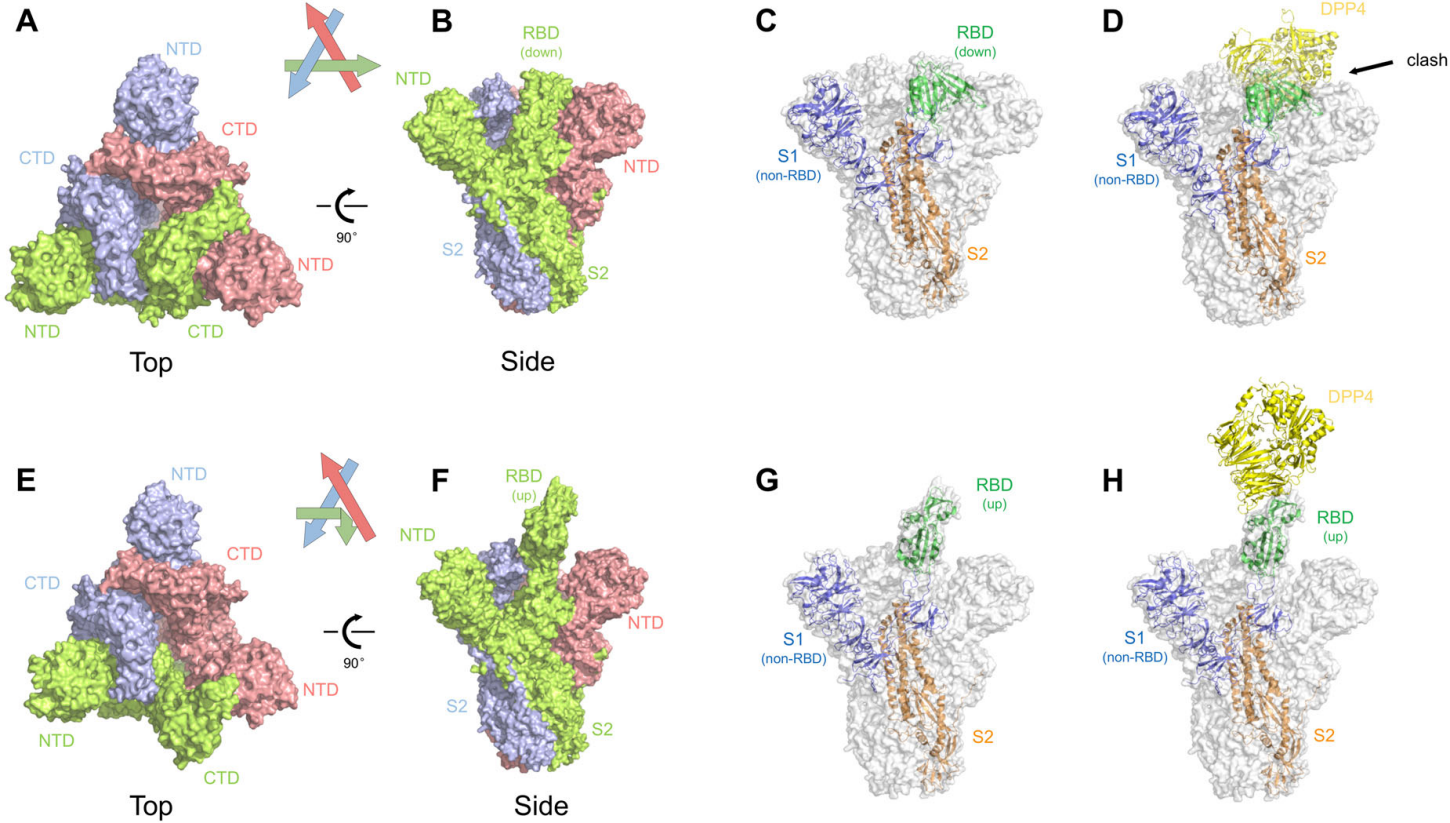
Structural insights of the MERS-CoV spike glycoprotein. (A–B) Top and side view of the MERS-CoV spike trimer with all RBD in ‘down' position, shown as molecular surface (PDB ID: 5W9J). The three protomers are coloured green, lightblue, and red, respectively. The labels are the same with those in Figure 1. (C) One of the three protomers in (B) is highlighted as cartoon representation whereas the other two protomers are faded in white. The RBD in ‘down' position is coloured in green. The non-RBD S1 region was coloured deep blue and the S2 region was coloured orange. (D) Superimposition of RBD-bound DPP4 (PDB ID: 4L72) into the MERS-CoV spike trimer. Clashes were observed between DPP4 and the other two S1 regions in the trimer structure. (E–F) Top and side view of the MERS-CoV spike trimer with one RBD in ‘up' position and the other two in ‘down' position, shown as molecular surface (PDB ID: 5W9H). Same colour codes are used as in (A–B). (G) The protomer with RBD in ‘up' position is highlighted as cartoon representation, with RBD in green, non-RBD S1 region in deep blue, and S2 in orange. (H) Superimposition of the RBD-bound DPP4 into the MERS-CoV spike trimer, with DPP4 interacting with the ‘up' RBD. No steric clash was observed.

Recently, structural studies on the prefusion state spike protein of MERS-CoV and SARS-CoV have provided more insights into the spike-mediated membrane fusion process [31–34]. The MERS-CoV spike protein trimerizes and folds into a metastable prefusion conformation on the virion surface, in which three S1 domains fold into a steady trimer structure and sit on top to stabilize the coiled S2 domains (Figure 2(A–B)). We and others have identified that the RBD of SARS-CoV and MERS-CoV can be found either buried (‘down' position) or exposed (‘up' position) in the spike trimer structure [31,33–35]. The two conformational states of RBD may have distinct roles during receptor binding and membrane fusion: only the RBDs in ‘up' position, but not those in ‘down' position, can bind to the cell receptor DPP4 (Figure 2(C–D)). Great steric clash was observed between DPP4 and neighboring spike protomers when we mapped it to the RBD in ‘down' position (Figure 2(C–D)). Transformation of the RBD from the buried to the exposed state is therefore a prerequisite for receptor binding (Figure 2(G–H)). On the other hand, this conformational change also seems to open up the stable cap structure sitting above the S2 cores (Figure 2(E–F)). This may lead to disassociation of S1 trimer and exposure of the fusion apparatus, triggering the membrane fusion process.

To gain a better understanding of MERS-CoV interaction with cellular receptors at atomic levels, we and others have determined the crystal structure of MERS-CoV RBD bound to the extracellular domain of its cellular receptor dipeptidyl peptidase 4 (DPP4) [23,24]. We showed that MERS-CoV RBD consists of a core and a receptor binding subdomain. MERS-CoV RBD and the related SARS-CoV RBD share a high degree of structural similarity in their core subdomains, but are notably divergent in the receptor binding subdomains [36]. The receptor binding subdomain of MERS-CoV RBD directly interacts with blades 4 and 5 of DPP4 propeller instead of its intrinsic hydrolase domain. The interface consists of a buried surface of ∼2550 Å^2^ involving 14 residues in receptor binding subdomain interacting with 15 residues in DPP4. The actual binding forces are mediated through two major binding patches. Patch 1 represents 49% of buried surface and forms between the C-terminal end of the long loop connecting the β6 and β7 strands and blade 4 of DPP4. Patch 2 occupies 51% of buried surface and forms a slightly concaved outer surface at the far end of the MERS-CoV receptor binding subdomain and a linker containing a short helix between blade 4 and blade 5 of DPP4. The concaved outer surface is made by the short β6 strand, C-terminal parts of β5 and β7 strands, N-terminal part of β8 strand and the β5-β6 linking loop. It is hoped that better understanding of the atomic details of the spike glycoprotein, as well as the interface between MERS-CoV RBD and DPP4 will provide the structural basis for rational design and development of therapeutics and vaccines against MERS-CoV infection.

## 3. Neutralizing monoclonal antibodies against MERS-CoV infection

Neutralizing antibodies are a major component of protective immunity against viral infection in humans. Polyclonal by nature, the antibody response in vivo mobilizes a dynamic and complex mixture of monoclonal antibodies (mAbs) that work in concert to target various antigenic domains on the viral envelope glycoprotein. Identifying the neutralizing mAbs that constitute the neutralizing activity of polyclonal response and their recognized antigenic domains has therefore become the first crucial step towards gaining a better understanding of the protective antibody response, developing clinical intervention methods, and designing immunogens capable of eliciting neutralizing antibodies.

**Figure 3.**
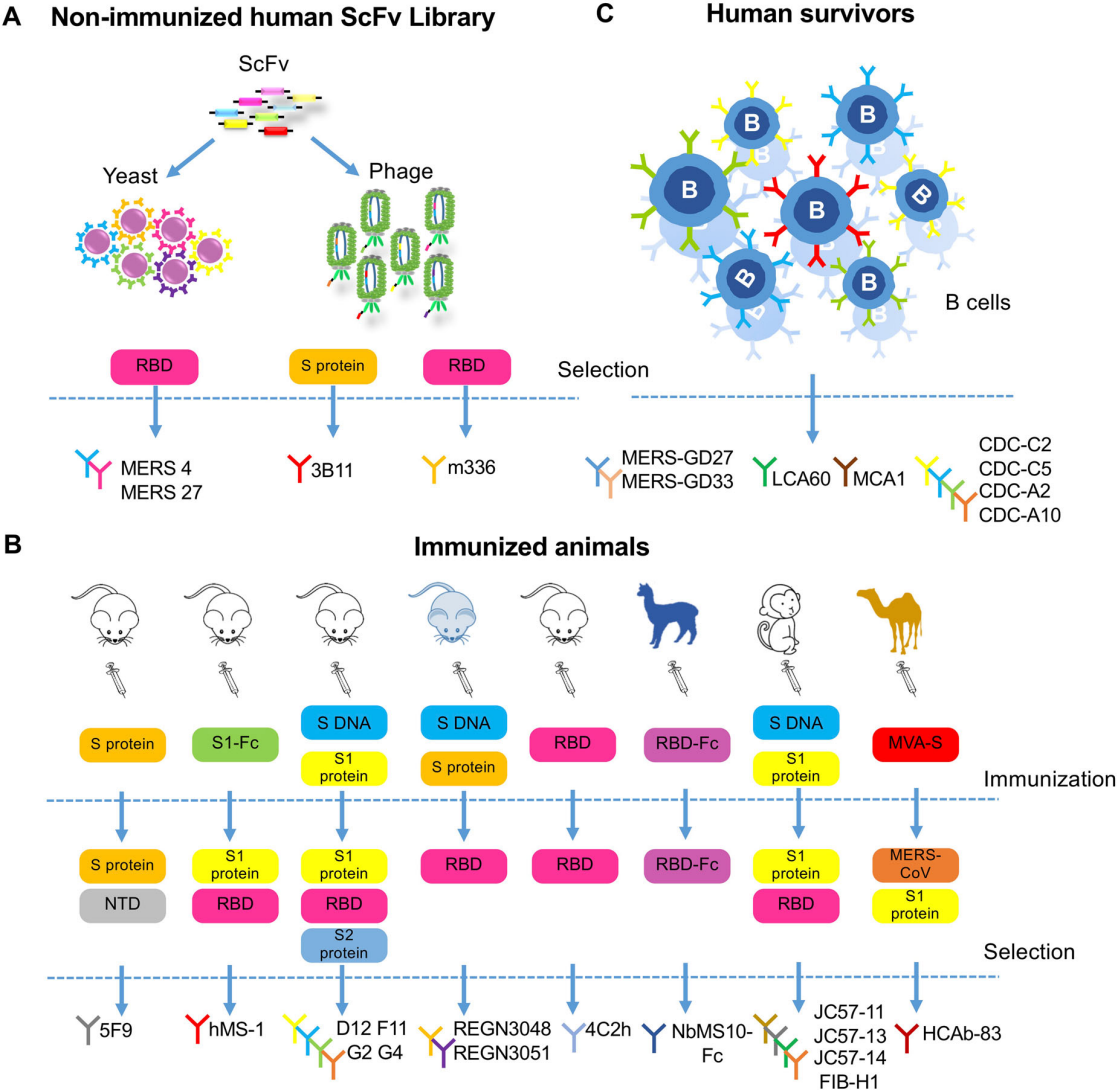
Development of monoclonal antibodies against MERS-CoV. (A) Monoclonal antibodies sorted from non-immunized human scFv (single-chain fragment variable) libraries. MERS-4 and MERS-27 were isolated from a non-immunized human scFv library displayed on yeast with MERS-CoV spike RBD as bait protein. Similarly, 3B11 and m336 were isolated from non-immunized human scFv phage libraries with MERS-CoV S protein or RBD protein as bait protein, respectively. (B) Monoclonal antibodies sorted from immunized animals. The antibodies 5F9, hMS-1 (Mersmab-1), D12, F11, G2, G4, 4C2h (4C2), REGN3048 and REGN3051 were isolated from mice immunized with the indicated vaccines labelled in the colour-coded boxes, each representing a different immunogen; the bait or target protein for antibody selection were also listed. The mice from which REGN3048 and REGN3051 were isolated were given the pale blue colour to indicate that they express human immunoglobulin genes. NbMS10-Fc, JC57-11, JC57-13, JC57-14, F1B-H1 and HCAb-83 were isolated from larger animal including llama, rhesus macaque and camels as indicated. The vaccines and selection criteria were also shown. NTD (N-terminal domain), Fc (fragment constant). (C) Monoclonal antibodies isolated from human survivors recovered from MERS-CoV infection. MERS-GD27, MERS-GD33, LCA60, CDC-C2, CDC-C5, CDC-A2 and CDC-A10 were generated by culturing B cells sorted from the patient and screening for MERS-CoV-specific antibodies. MCA1 was produced by constructing a phage library displaying scFv cloned from a convalescent patient.

Great achievements have been made in the isolation of neutralizing mAbs in the past few years using various technology platforms (Figure 3). Up till now, more than 20 mAbs, most of which are human or humanized antibodies, have been described by scientists from all over the world. These antibodies are listed in chronological order of publication in Table 1, together with their unique biochemical and antiviral properties against MERS-CoV infection observed in cell culture and experimental animal models.

**Table 1.** Advancement in MERS-CoV monoclonal antibodies development.

Name	Source ‡	Target	Potency and Binding †			Evaluation Platforms#	Mechanism	Ref
			*IC_50_ Pseudo* (μg/ml)	*IC_50_ live* (μg/ml)	*K_d_* (nM)			
MERS-4	Non-immune human ScFv (yeast library)	RBD	0.053	0.48	0.98 (RBD)	In vitro	Group 3	[37–39]
MERS-27		RBD	9.21	1.92	71.2 (RBD)	In vitro	Group 1	

3B11	Non-immune human ScFv (phage library)	RBD	3.50	N.A.	0.057 (S1)	NHP (Prophylactic)		[40,41]

m336	Non-immune human ScFv (phage library)	RBD	0.005	0.07	0.099 (RBD)	hDPP4-Tg mice (Prophylactic & post-exposure) Rabbit (Prophylactic) NHP (Post-exposure)	Group 2	[42–46]

hMS-1	Mice immunized with S1-Fc (antibody humanized)	RBD	0.089	3.34	0.045 (RBD)	hDPP4-Tg mice (Post-exposure)		[47,48]

D12	Mice immunized with S-DNA & S1	RBD	0.013	N.A.	9.93 (RBD) 6.63 (S1)	In vitro	Group 1	[49,50]
F11		RBD	0.008	N.A.	114 (RBD) 3.49 (S1)	In vitro		
G2		S1 (non-RBD)	0.013	N.A.	1.69 (S1)	hDPP4-Tg mice (Prophylactic)		
G4		S2	0.133	N.A.	8.65 (S2)		S2	

REGN3048	Humanized mice immunized with S-DNA & S	RBD	0.009	0.026	0.048 (RBD)	hDPP4-KI mice (Prophylactic & post-exposure) NHP (Prophylactic)		[51,52]
REGN3051		RBD	0.010	0.066	0.043 (RBD)			

LCA60	Human Survivor	RBD	0.010	0.150	0.12 (S)	Ad5-hDPP4 mice (Prophylactic & post-exposure) NHP (Prophylactic)		[53,54]

4C2h	Mice immunized with RBD (antibody humanized)	RBD	1.8	6.25	217 (RBD)	Ad5-hDPP4 mice (Prophylactic & post-exposure)	Group 1	[55]

MCA1	Human Survivor	RBD	N.A.	0.39	N.A.	NHP (Prophylactic & post-exposure)	Group 2	[56]

5F9	Mice immunized with S	S1 (NTD)	0.24	0.2	5.42 (NTD)	In vitro		[57]

CDC2-C2	Human Survivor	RBD	0.0057	0.058	N.A.	hDPP4-Tg mice (Prophylactic)	Group 2	[50]
CDC2-A2		S1 (non-RBD)	0.2180	0.024	N.A.	In vitro		
CDC2-A10		S1 (non-RBD)	0.0268	0.032	N.A.	In vitro		
JC57-14	NHP immunized with S-DNA & S1	RBD	0.0084	0.07	N.A.	In vitro	Group 1	
JC57-13		S1 (non-RBD)	0.0085	<0.0032	N.A.	In vitro		
FIB-H1		S1 (non-RBD)	0.0083	N.A.	N.A.	In vitro		

MERS-GD27	Human Survivor	RBD	0.0010	0.001	0.78 (S)	hDPP4-Tg mice (Prophylactic & post-exposure)	Group 2	[58,59]
MERS-GD33		RBD	0.0013	0.001	0.58 (S)	In vitro		

NbMS10-Fc	Llama immunized with RBD (nanobody humanized)	RBD	N.A.	2.33	0.35 (S1)	hDPP4-Tg mice (Prophylactic & post-exposure)		[60]

HCAb-83	Camel immunized with MVA-S (nanobody humanized)	RBD	N.A.	0.0014	0.103 (S)	hDPP4-Tg mice (Prophylactic)		[61]

‡ RBD, S, S1, and S1-Fc are all recombinant proteins. Modified Vaccinia Ankara (MVA).

† The representative mAbs are chosen if there are multiple antibodies in the same panel. These data are directly copied from original publications. Data listed here are for full-length human IgG formats of the antibody, or the human Fc-conjugated format for the nanobodies. Target protein for binding affinity tests are indicated in the parenthesis in the K_d_ column. Abbreviation: 50% inhibitory concentration (IC_50_), equilibrium disassociation constant (K_d_), data not available (N.A.).

# Abbreviations for evaluation platforms: human DPP4 transgenic (hDPP4-Tg) mice with global/epithelial hDPP4 expression, human DPP4 knock-in (hDPP4-KI) mice with hDPP4 replacing mDPP4 in situ, mice transduced with human adenovirus 5 vector expressing hDPP4 (Ad5-hDPP4 mice), and non-human primates (NHPs).

It is apparent that the single chain fragment variable (scFv) library approach allows rapid discovery of mAb, without time constraints from immunizing experimental animals or approaching convalescent individuals of MERS-CoV infection. The earliest mAbs reported in 2014 were identified through screening non-immune human scFv libraries with the ectodomain of S glycoprotein (mAb 3B11) [40] or soluble RBD from S glycoprotein (MERS-4, MERS-27 and the m336 panel) [37,42] as bait protein (Figure 3(A)). These antibodies all demonstrated high neutralizing activities and therefore were widely used as reference antibodies in later studies.

Antibodies have also been generated from immunized animals (Figure 3(B)). Several groups have reported mAbs isolated from either wild-type inbred mice or transgenic mice expressing human antibody-variable heavy chains and κ light chains. Mersmab-1 (known as hMS-1 after humanization) was isolated from mice immunized subcutaneously with chimeric S1-Fc [47,48]. The mAbs 2E6 and 4C2 (humanized form 4C2 h) were isolated in mice immunized with recombinant RBD produced in insect cells [55]. Furthermore, two human-like mAbs, REGN3048 and REGN3051, were directly cloned from transgenic mice expressing human versions of the antibody after immunization with DNA encoding S glycoprotein and purified recombinant S glycoprotein [51]. Both mAbs have been tested in humanized mice models and in non-human primates [51,52]. The authors indicated that the advantages of their system not only lay in the human component of their antibodies but also in the quick speed associated with isolation and production, since no humanization or optimization step was required. Currently, REGN3048 and REGN3051 have entered phase I clinical trials.

Most of the mAbs reported so far target the RBD region of S glycoprotein, but RBD does not seem to be the only target for anti-MERS-CoV antibody responses. Recently, a mAb targeting the S1 N-terminus domain (NTD) region, which does not contain RBD, was isolated from mice immunized with S glycoprotein [57]. This antibody, 5F9, was shown to successfully block virus entry in cell culture models and the efficacy was comparable to other mAbs in IC_50_. Further, the mAb panel D12, F11, G2 and G4 were generated by priming mice with DNA encoding the full-length S glycoprotein and boosting them with S1 protein. Among them are two mAbs that target the non-RBD S1 (mAb G2) and S2 region (mAb G4), respectively [49]. These non-RBD-binding antibodies potently neutralized pseudo- and live MERS-CoV in cell culture and were also protective in mouse models [49,50]. Together, the development of these antibodies elucidates that RBD may not be the single target for anti-viral antibody response. More studies are needed to elaborate the detailed mechanisms for these antibodies.

Apart from the traditional approach of isolating mAbs from immunized mice, several groups have turned to larger animal models for antibody isolation. One group immunized rhesus macaques with combined DNA and protein vaccines and isolated a panel of mAbs, including JC57-11, JC57-13, JC57-14, and FIB-H1, targeting both RBD and non-RBD S1 region of the S glycoprotein, all with potent neutralizing activities [50]. Another group immunized llama with recombinant RBD and screened the nanobody library for high-affinity single heavy chain antibody (nanobody) against RBD. The humanized form NbMS10-Fc was constructed by combining the variable domain of the nanobody with the human constant Fc domain, and it was shown to protect mice from lethal MERS-CoV challenge [60]. Similarly, Stalin *et al* isolated a nanobody targeting RBD from camels immunized with MVA encoding S glycoprotein. The humanized form HCAb-83 has high binding affinity to S protein and potent neutralizing activities to live virus [61]. These nanobody-derived mAbs are smaller in molecular weight and more stable than traditional antibodies, and may provide a new option for future antibody isolation.

In terms of closeness to authentic human antibodies, no approach can compete with those based on direct B cell cloning from convalescent individuals. One such mAb LCA60 was isolated from memory B cells of human survivors of MERS-CoV infection and was among the most potent mAbs reported in neutralizing pseudo- and live viruses [53]. More mAbs isolated from human survivors were described as more convalescent blood samples became available, including MCA1 [56], CDC-C2, CDC-C5, CDC-A2, CDC-A10 [50], MERS-GD27, and MERS-GD33 [58,59] (Figure 3(C)), all with potent neutralizing activities against MERS-CoV. The mAbs LCA60, CDC-C2, MCA1, and MERS-GD27 were also tested to be protective in animal models.

As MERS-CoV research progressed quickly in the past few years, many mAbs have been tested for prophylactic or therapeutic protection efficacy in human DPP4 transgenic / transduced mice models, and a few have entered large animal model trials such as in rabbits or non-human primates (NHPs). However, as different animal models were established among labs worldwide with slightly different evaluation end points, it is difficult to make a direct comparison among these mAbs. This is also true for *in vitro* evaluation of neutralizing activities – since different cell lines, pseudo-viruses, and neutralizing assay techniques are utilized, the published IC_50_ values can only serve as indirect reference for comparison. Head to head comparison in the same experimental system would be required to identify the most protective mAb or combination of mAbs against MERS-CoV infection in order to proceed to clinical trials.

## 4. Structure features of neutralizing mAbs against MERS-CoV infection

**Figure 4.**
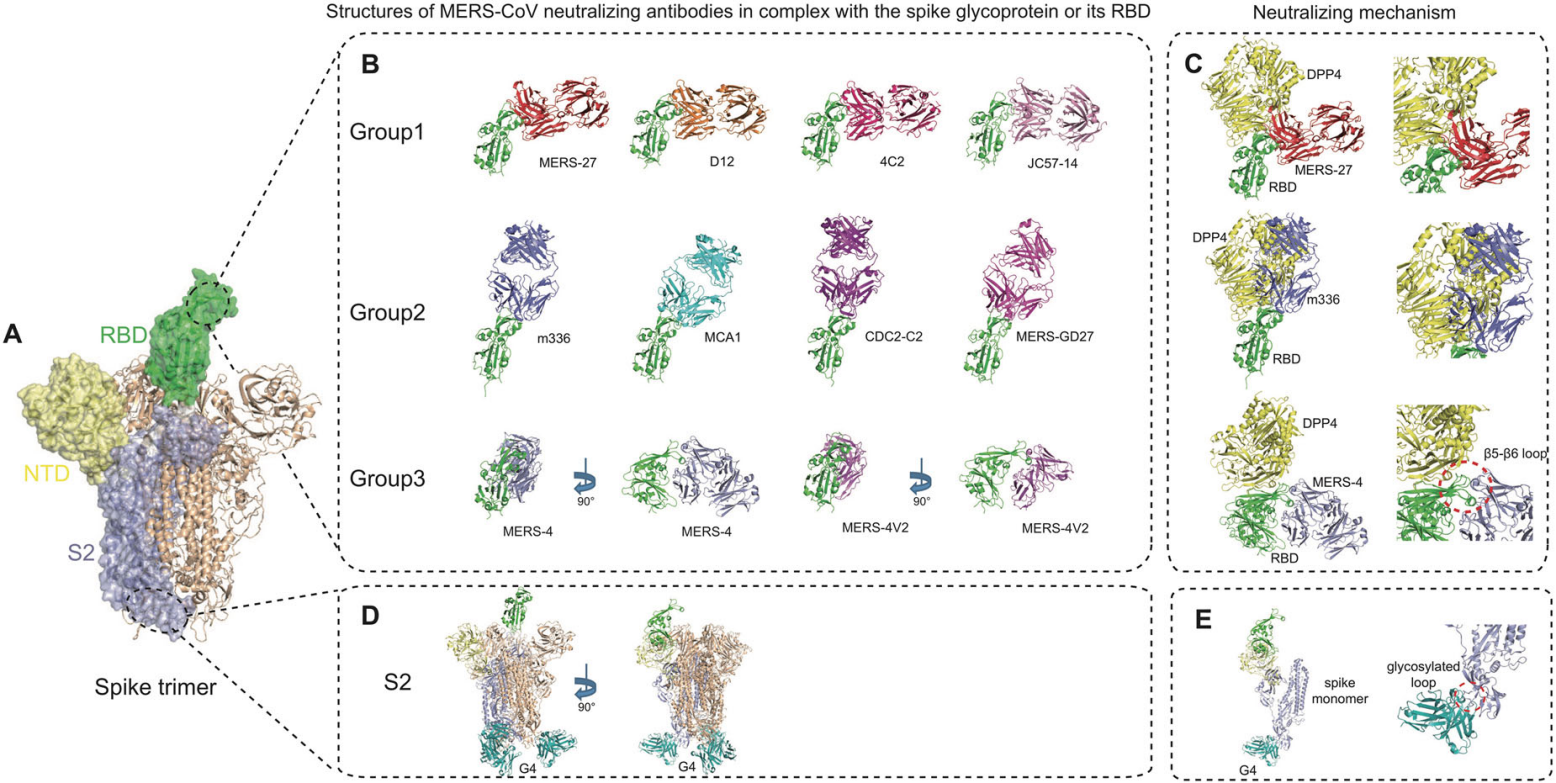
Advancement in structural studies of MERS-CoV neutralizing monoclonal antibodies. (A) Structure of MERS-CoV spike trimer ectodomain (PDB ID: 5X5F). A single protomer of the trimeric spike protein with RBD in ‘up' conformation is shown as molecular surface. The RBD, NTD and S2 subunit are coloured in green, paleyellow and lightblue, respectively. The two remaining protomers with RBD in ‘down' conformation are shown in cartoon representation and coloured in wheat. (B) Structures of MERS-CoV neutralizing antibodies targeting RBD. Antibodies are classified into three groups and shown as cartoon representation. The RBD is coloured in green and antibodies in different colours. Group 1 includes MERS-27 (PDB ID: 4ZS6), D12 (PDB ID: 4ZPT), 4C2 (PDB ID: 5DO2) and JC57-14 (PDB ID: 6C6Y). Group 2 includes m336 (PDB ID: 4XAK), MCA1 (PDB ID: 5GMQ), CDC2-C2 (PDB ID: 6C6Z) and MERS-GD27. Group 3 includes MERS-4 (PDB ID: 5ZXV) and MERS-4V2 (PDB ID: 5YY5). (C) Neutralizing mechanisms of MERS-CoV neutralizing antibodies targeting RBD. The left panel shows the structural superimposition of the representative antibodies from the three groups (MERS-27, m336 and MERS-4) and DPP4 (coloured in yellow) bound to RBD (coloured in green, PDB ID: 4L72) at the same time. The right panel is enlarged view of steric clashes between the antibodies and the DPP4 and a significant conformational difference in the RBD β5-β6 loop between antibody-bound and DPP4-bound states. (D) Structure of G4 fab (coloured in teal) in complex with spike trimer (PDB ID: 5W9H). (E) Side views of spike monomer bound to G4 fab. The enlarged view of the glycosylated loop in the S2 subunit recognized by G4 is shown on the right.

We and others have carried out structural studies of MERS-CoV neutralizing antibodies in complex with MERS-RBD to understand neutralizing mechanism at atomic levels (Figure 4). Based on the epitopes revealed by structural studies, MERS-CoV antibodies targeting RBD can be classified into three groups (Figure 4(B), Table 1).

The first group consists of antibodies MERS-27, D12, 4C2 and JC57-14, which interact with the C-terminal segment of the β6-β7 loop and β7 strand of RBD by both heavy and light chains (Figure 4(B)) [38,49,50,55]. Their common epitopes on the RBD include residues Val527, Ser528, Ile529, Val530, Pro531, Ser532, Trp535, Glu536 and Asp539 in the β6-β7 loop. The residues Trp535, Glu536 and Asp539 also happen to be within the DPP4-binding site patch 1 of MERS-CoV RBD [23], mediating interaction with Lys267 and the carbohydrate moiety linked to Asn229 of DPP4 [38]. Therefore, the Group 1 antibodies would directly compete with DPP4 in binding to RBD by interfering with both protein–protein and protein–carbohydrate interactions between RBD and DPP4. Structural super-impositions also showed that these four antibodies and DPP4 would have steric clashes between the variable domain of the heavy chain and the propeller domain of DPP4 if they simultaneously bind to RBD (Figure 4(C)).

The second group consists of antibodies m336, MCA1, CDC2-C2 and MERS-GD27, which interact with the β5-β8 strands, β5-β6 loop and β6-β7 loop in RBD mainly by the heavy chain (Figure 4(B)) [43,50,56,58]. Their common epitope consists of Phe/Leu506, Asp510, Trp535, Glu536, Asp539, Tyr540, Tyr541, Arg542, and Trp553. Although antibodies in both Group 1 and Group 2 share the binding residues Trp535, Glu536 and Asp539, their approaching angles to the RBD are significantly different. As shown in Figure 4(C), the approaching angle of Group 2 antibodies is closer to that of DPP4 by rotating approximately 90 degrees anti-clockwise from that of Group 1 antibodies, thereby generating more steric clashes with DPP4. This is also evidenced by a larger overlap between the common epitope of Group 2 antibodies and DPP4-binding site on RBD [23]. As a representative of Group 2 antibodies, m336 exhibits very potent neutralizing activity by not only mimicking critical interactions between RBD and DPP4 but also adopting an approaching angle similar to that of DPP4 (Figure 4(C)).

The third group consists of antibody MERS-4 and its variant MERS-4V2 with four residue replacements in the HCDR3 (Figure 4(B)) [39]. By structural determination, it was shown that MERS-4 Fab and MERS-4V2 scFv share the same mode of binding to the RBD (Figure 4(B)) [39]. Analysis of the RBD/MERS-4V2 complex structure showed that the antibody contacts with the β5-β6, β6-β7 and β7-β8 loops of the receptor-binding subdomain in RBD [39]. The epitope involves Leu507, Ser508, Gln516, Asn519, Asn521, Gln522, Tyr523, Pro525, Lys543, Leu545, and Gly550 [39]. To be note, the MERS-4 epitope has no overlap with DPP4-binding site (Figure 4(C)). By approaching the RBD outside the DPP4-binding site, MERS-4 recognizes a unique epitope different from all previously reported RBD-targeting antibodies. Comparisons of RBD in DPP4-bound and MERS-4-bound states revealed that binding of MERS-4 induces or fixes the β5-β6 loop into a conformation in which it folds into a shallow groove on the RBD interface critical for accommodating a short helix of DPP4, thereby indirectly disrupting the interaction between RBD and DPP4 (Figure 4(C)). Such different epitope and mechanism enable MERS-4 to synergize with other antibodies including RBD-targeting MERS-27 and m336 in neutralization, which provides valuable addition for the combined use of antibodies against MERS-CoV infection [39].

In addition to the aforementioned ten antibodies targeting RBD, the near atomic resolution cryo-EM structures of the trimeric MERS-CoV spike and its complex with antibody G4 were also determined (Figure 4(D)) [33]. G4 is the first reported S2-targeting antibody and its epitope consists of a glycosylated, solvent-exposed loop residing in a connector domain between the HR1 and HR2 of the S2 subunit. In the unbound spike trimer structure this loop is largely disordered, whereas it extends out from two β-strands and is surrounded by all six CDRs (complementarity determining regions) of the mAb G4 upon antibody recognition (Figure 4(E)). The specific spike-G4 interaction may stabilize the loop and further impede conformational changes of S2 subunit essential for membrane fusion after DPP4 binding. The binding epitope for G4 in S2 subunit is more conserved than RBD among MERS-CoV isolates, shedding light on G4 as a potential broad-spectrum neutralizing antibody for MERS-CoV. Yet this loop between HR1 and HR2 is variable in sequence and length among different viruses even in lineage C betacoronaviruses [33], limiting its application to other coronaviruses. In terms of pan-coronavirus medical countermeasures (MCMs), the recently developed fusion inhibitor peptide EK1 is a potential candidate. The peptide EK1 was designed to target the more conserved HR1 region of the S2 stem, and was shown to block cell–cell fusion induced by spike protein from multiple human coronaviruses [30].

In general, most reported MERS-CoV neutralizing antibodies recognize the RBD in the S1 subunit, and these antibodies are highly potent in neutralization. These facts show that the RBD in the S1 subunit is a major vulnerable site for antibody recognition and neutralization. To be note, the RBD is also the region where most naturally occurring mutations of the S glycoprotein occur. Currently, the comprehensively studied antibodies targeting the non-RBD region of the spike glycoprotein also include mAb 5F9 targeting the N-terminal domain (NTD) of the S1 subunit [57], as well as mAbs G2, CDC2-A2, CDC2-A10, JC57-13 and FIB-H1 targeting the non-RBD region of the S1 subunit [33,50]. However, the detailed epitopes and specific mechanisms are still unclear for these antibodies. We expect that more antibodies with new neutralizing epitopes and/or mechanisms would be important for the combined use of antibodies against MERS-CoV infection.

## 5. Advancement in MERS-CoV vaccine development

Although monoclonal antibodies show promising anti-viral effects in both cell culture and animal models against MERS-CoV infection, their roles are still limited in large-scale disease prevention in MERS-CoV high risk areas, as the therapeutic window is generally narrow for mAbs and mass-scale production is time- and resource-consuming. Vaccines still remain the best choice for MERS-CoV prevention.

Given its critical role in mediating viral entry and as major targets for neutralizing antibodies, S glycoprotein and its RBD have become the prime targets for MERS-CoV immunogen design and vaccine development. Various approaches have been applied and more than twenty vaccine candidates have been reported in the past few years, including vaccines based on inactivated virions [62,63], virus-like particles [64], recombinant viral vectors [65–80], DNA [49,81,82], recombinant protein subunits [33,49,83–92], and nanoparticles [80,93,94]. Table 2 summarizes the critical features of these approaches and their protective potentials in experimental animal models.

**Table 2.** Advancement in MERS-CoV vaccine development.

Vaccine platform	Composition §			Immunization strategy‡			Animal Model#	Efficacy*	Ref
				* Schedule *	* Route *	* Dosage *			
** MERS-CoV **	Inactivated		EMC/2012	2 doses (3 weeks interval)	i.m.	1×10^6^ TCID_50_ Alum / MF59	hDPP4-Tg mice	nAb↑ Viral Load ↓	[62]
			EMC/2012	3 doses (4 weeks interval)	i.m.	1 µg S (equivalent) Alum + CpG	Ad5-hDPP4 mice	nAb↑(against RBD) Viral Load ↓ Pathology ↓	[63]
	Virus like particle		Alum	4 doses (2 weeks interval)	i.m.	250 μg VLPs 250 μg Alum	NHPs	nAb↑ Cellular Immunity ↑	[64]

** Viral Vector Based **	MVA		S	2 doses (3 weeks interval)	i.m./s.c.	1 × 10^8^ PFU	Ad5-hDPP4 mice	nAb↑ Cellular Immunity ↑ Viral Load ↓ Pathology ↓	[65,66]
			S	2 doses (4 weeks interval)	i.n. i.m.	2×10^8^ PFU (i.n.) + 1 × 10^8^ PFU (i.m.)	Dromedary Camel	nAb↑(against S1) Viral Load ↓ Pathology ↓	[67]
	Adenovirus		Ad5-S/S1	2 doses (week 0 i.m.+ week 3 i.n.)	i.m. i.n.	1×10^11^ vp	BALB/c mice	nAb ↑ (against S)	[68]
			Ad5-S	1 dose	i.m./ i.g.	1×10^9^ vp	BALB/c mice	nAb ↑ (against RBD) Cellular Immunity ↑ (i.m.)	[69]
			Ad41-S			5×10^9^ vp			
			ChAdOx1-S	1 dose	i.n./i.m.	1×10^8^ IU	hDPP4-Tg mice	nAb↑ Cellular Immunity ↑ Viral Load ↓ Pathology ↓	[70,71]
			Ad5-S1-CD40L	2 doses (4 weeks interval)	i.m.	1×10^9^ PFU	hDPP4-Tg mice	nAb↑ Viral Load ↓ Pathology ↓	[72]
			AdC68-S	1 dose	i.n.	2×10^9^ vp	hDPP4-KI mice	nAb↑ Cellular Immunity ↑ Viral Load ↓ Pathology ↓	[73]
	Measles Virus		S	2 doses (4 weeks interval)	i.p.	1×10^5^ TCID_50_	Ad5-hDPP4 mice	nAb↑ Cellular Immunity ↑ Viral Load ↓ Pathology ↓	[74,75]
	VEEV Replicon Particle		S	2 doses (4 weeks interval)	foot-pad	1×10^5^ IU	Ad5-hDPP4 mice hDPP4-Tg mice	Viral Load ↓	[76,77]
	VSV-ΔG		S	1 dose	i.n./i.m.	2 × 10^7^ FFU	NHPs (also in mice)	nAb↑ Cellular Immunity ↑	[78]
	RABV		S1	3 doses (1-2 weeks interval)	i.m.	10 µg inactivated virus	Ad5-hDPP4 mice	nAb ↑ Viral Load ↓	[79]

** Viral vector + nanoparticle **	Ad5-S + Nanoparticle(S)			1×Ad5-S 2×nanoparticle (2-3 weeks interval)	i.m.	1×10^9^ IU 5 µg S + Alum	BALB/c mice	nAb↑ Cellular Immunity ↑ Pathology ↓	[80]
** DNA **	S (consensus sequence)			3 doses (3 weeks interval)	i.m.	0.5-2 mg	NHPs (also in mice and camels)	nAb↑ Cellular Immunity ↑ Viral Load ↓ Pathology ↓	[81]
	S1 (1-725)			3 doses (3 weeks interval)	i.m.	0.1 mg	Ad5-hDPP4 mice	nAb↑ Cellular Immunity ↑ Viral Load ↓	[82]

** DNA + protein **	S DNA + S1 Protein			2×DNA 1×Protein Boost (4 weeks interval)	i.m.	1 mg DNA 100 μg Protein	NHPs (also in mice)	nAb ↑ Pathology ↓	[49]

** Protein Subunit **	RBD-Fc (377-588)	MF59		3 doses (3 weeks interval)	s.c.	1–10 μg	Ad5-hDPP4 mice	nAb↑ Cellular Immunity ↑ Viral Load ↓	[83–86]
		Alum		2 doses (4 weeks interval)	s.c.	5 μg	hDPP4-Tg mice	nAb ↑ Pathology ↓	[87]
	RBD trimer (377-588)	Alum		2 doses (4 weeks interval)	i.m.	5 μg	hDPP4-Tg mice	nAb ↑ Pathology ↓	[88]
	RBD-Fc (377-662)	Poly(I:C)		5 doses (Week 0, 3, 6, 12, 24)	i.n.	10 μg	BALB/c mice	nAb ↑ (against RBD)	[89]
	RBD (367-606)	Alum		3 doses (Week 0, 8, 25)	i.m.	200 + 2×100μg 50 + 2×25μg	NHPs (also in mice)	nAb↑ Cellular Immunity ↑ Viral Load ↓ Pathology ↓	[90,91]
	NTD (18-353)	Alum + CpG		3 doses (4 weeks interval)	i.m.	10 μg	Ad5-hDPP4 mice	nAb↑ Cellular Immunity ↑ Pathology ↓	[92]
	S prefusion trimer	Sigma adjuvant		2 doses (3 weeks interval)	i.m.	10 µg	BALB/c mice	nAb↑	[33]

** Nanoparticle **	S			2 doses (3 weeks interval)	i.m.	1–10 μg Spike	Ad5-hDPP4 mice	nAb ↑ Viral Load ↓	[93,94]

§ Composition indicates specific virus strain, truncation of DNA / protein, or adjuvants used in the vaccine design. Modified Vaccinia Ankara (MVA), Venuzuelan Equine Encephalitis Virus (VEEV), Vesicular Stomatitis Virus without G protein (VSV-ΔG), Rabies virus (RABV), tissue plasminogen activator (tPA). S indicates full length Spike Glycoprotein with the transmembrane domain (TM).

‡ Abbreviations for vaccination route: intramuscular (i.m.), intranasal (i.n.), subcutaneous (s.c.), intragastric (i.g.), and intraperitoneal (i.p.).

Different units are applied to describe doses in each platform: plaque forming units (PFU), virus particle (vp), half-tissue-culture-infectious-dose (TCID_50_), and infectious units (IU).

# Abbreviations for animal models: human DPP4 transgenic (hDPP4-Tg) mice with global/epithelial hDPP4 expression, human DPP4 knock-in (hDPP4-KI) mice with hDPP4 replacing mDPP4 in situ, mice transduced with human adenovirus 5 vector expressing hDPP4 (Ad5-hDPP4 mice), and non-human primates (NHPs).

* Efficacy in the specific animal model listed in the previous column. If studies are conducted in multi-animal models, only results in the highest-level model are shown. Neutralizing antibody (nAb). ↑ indicates more, while ↓ indicates less.

Up till now, only two vaccine candidates, GLS-5300 and MERS001, have entered human clinical trials. The vaccine GLS-5300 was the first to be tested in healthy human volunteers. It is a DNA plasmid encoding the MERS-CoV S glycoprotein, requiring two-to-three injections delivered by electroporation [81]. The phase I clinical trial was started in 2016 at the Walter Reed Army Institute, and another phase I/II clinical trial is being conducted in Korea to test dosage safety and immunogenicity. Another vaccine candidate, MERS001, is a replication-deficient chimpanzee adenovirus (ChAdOx1) containing the MERS-CoV S glycoprotein antigen [70,71]. This vaccine only requires one-time administration of 5×10^9^–5×10^10^ virus particles via intramuscular route, and the local adverse events as well as immunogenicity will be evaluated in the phase I clinical trial conducted at the University of Oxford. In addition, one more candidate vaccine has been tested in dromedary camels either for potential human use or straight into veterinary use. It explores a modified vaccinia virus Ankara (MVA) as a vector to express MERS-CoV S glycoprotein [67]. The regimen involves immunization through intranasal as well as intramuscular routes twice at a 4-week interval. The vaccinated camels demonstrated a significant reduction of excreted infectious virus and viral RNA transcripts in vaccinated animals upon MERS-CoV challenge. Protection against MERS-CoV infection correlated with the presence of serum neutralizing antibodies to MERS-CoV. As MVA has established a reasonably good safety profile in humans and induced desirable protective immunity in camels, it represents one of the potential candidates to be further evaluated in humans in the near future.

The remaining vaccine candidates are all in the stages of preclinical or laboratory development and invariably target the S glycoprotein or RBD critical for viral entry (Table 2). Vaccines based on inactivated [62,63] or virus-like particles [64] have historical precedence in inducing protective immune responses in humans. Whether the same strategies are applicable to MERS-CoV requires further studies, particularly when it comes to possible safety concerns [62].

Apart from MERS001 and the MVA-based vaccine tested in dromedary camels, other vector-based approaches are also being actively pursued, including adenovirus [68,69,72,73,80], measles virus [74,75], VEEV replicon particle [76,77], vesicular stomatitis virus [78], and rabies virus [79]. All recombinant viruses encoding the MERS-CoV S or S1 antigen demonstrated strong immunogenicity in mice or non-human primate models, and some were shown to confer protection in MERS-CoV challenge mouse models (Table 2). However, concerns remain regarding the pre-existing immunity against these viral vectors from natural infection, because it would diminish the vaccine potency [95]. To overcome the issue of pre-existing immunity against human adenoviruses while preserving their advantages such as high yields and strong immunogenicity, rare serotypes of chimpanzee adenovirus of low human seroprevalence may be adopted as viral vectors [70,73]. Our group recently developed a vaccine candidate with replication-defective chimpanzee adenovirus C68 (AdC68) vector expressing full length MERS-CoV S glycoprotein. Seroprevalence of AdC68 is around 2% in human population, much lower than that of the commonly used human adenovirus 5 (HuAd5) vector (>60%) [96,97]. One intra-nasal administration of 2 × 10^9^ viral particles completely protected human DPP4 knock-in (hDPP4-KI) mice from lethal MERS-CoV challenge, and passive transfer of AdC68-S immune sera conferred survival advantage in lethal challenge mouse models [73]. Further, the safety profiles of these vectors have yet to be extensively tested in humans. Recently, Hashem and colleagues showed that the adenovirus-based S1 vaccine may pose potential safety concerns because it may induce pulmonary perivascular hemorrhage in a MERS-CoV challenge mouse model, regardless of the its full protection upon lethal viral infection. They also showed that the pulmonary pathology can be mitigated by incorporating CD40L, an immune-modulator therefore potential molecular adjuvant, into the recombinant adenovirus-based vaccine [72]. Whether this vaccine-associated pathology is related to residual infectious viruses or unbalanced immune responses awaits further investigation. With this in mind, all future MERS-CoV vaccine candidate designs should take extra cautions on safety evaluation.

Furthermore, recombinant-protein-based vaccines are widely pursued. Strategies to solubilize the MERS-CoV S glycoprotein in order to form stable immunogens include forming nanoparticles and using soluble protein truncations. In particular, both nanoparticles formed with full length MERS-CoV S glycoprotein [93,94] and subunit RBD-based vaccines [83–90] have been shown to induce virus neutralizing anti­bodies and to protect mice when challenged with MERS-CoV. One RBD subunit vaccine also conferred protection in rhesus macaques [91]. This indicates that RBD alone as antigen may be sufficient for protective immunity to develop against the virus. Along with the finding that mAb targeting NTD is able to neutralize MERS-CoV, Lan *et al* showed that three doses of intramuscularly administered recombinant NTD protein also induced protective immunity against live MERS-CoV in human DPP4 transduced mouse model (Ad5-hDPP4 mice) [92]. More recently, with the structural insights into the spike glycoprotein, Pallesen *et al* developed a prefusion-stabilized S trimer vaccine by substituting proline residues into the S2 domain [33]. The introduction of proline disfavours the refolding of the linker between HR1 and the central helix, thus preventing the transition of spike into the post-fusion state. This rationally designed antigen, MERS S-2P, was shown to induce broader and more potent neutralizing activity than wild type spike trimer protein [33].

Finally, a prime-boost strategy based on a full-length S glycoprotein DNA vaccine followed by an S1-glycoprotein boost was able to induce virus-neutralizing anti­bodies and confer protection against the clinical severity of diseases in non-human primate models [49]. Compared with the protein-only regimen, the combination of DNA and protein induced a more functionally diverse antibody repertoire and stronger Th1 immune response. It was suggested that the native S glycoprotein conformation, formed on the cell surface after DNA vaccination, helped induce more diverse antibodies against MERS-CoV.

As summarized in Table 2, most of the aforementioned strategies require multiple immunizations which may pose additional logistic hurdles at the end point use. It is unclear whether these immunization strategies were empirically designed or due to relatively poor immunogenicity of candidate vaccines. For practical and compliant purposes, a single immunization with the highest immunogenicity in animals and humans will be preferred.

## 6. Conclusion

The outbreak of MERS-CoV in Saudi Arabia in 2012 reminded us of the 2003 SARS-CoV outbreak in China. Despite the differences in geographic location, epidemiology and immediate animal reservoirs, these two viruses share remarkable similarity in causing severe respiratory syndrome, leading to high fatality in humans and trigger serious public health concerns. With the advent of modern techniques in virology, immunology and vaccinology, we have gained substantial insights into the biology of MERS-CoV, and its pathogenesis with unprecedented speed and accuracy. As summarized in the current review, tremendous progress has been made in understanding (1) the entry process of MERS-CoV into target cells, (2) the structure and function of S glycoprotein and cellular receptor DPP4 in mediating viral entry, (3) antibody response during natural infection and isolation of broad and potent neutralizing mAbs, and (4) design and development of vaccine candidates using various innovative technologies. However, our progress in translating these discoveries into clinical application has been slow. Only two vaccine candidates and one mAb panel have entered phase I clinical trials for safety. Ironically, no vaccines and treatment strategies have been approved for SARS-CoV infection even after more than a decade of outbreak. We could not imagine how catastrophic it would be should SARS-CoV hit again or MERS-CoV continues to probe and gain strong capacity in transmission to and among humans.

We are facing a difficult predicament when it comes to public health challenges in the new era of emerging and re-emerging infectious diseases. On one hand, the human population is becoming ever mobile and exposed to an increasing number of pathogens. On the other hand, translating basic discoveries into preventative and treatment applications has been exceedingly slow. Among many plausible reasons, a lack of incentives in financial returns perhaps stands the tallest. The deadlock is not just happening to MERS-CoV and SARS-CoV but also to many other infectious pathogens such as Ebola, Marburg, Lassa, highly pathogenic avian influenza, HIV-1, and so on. Fundamental and drastic changes have to be made in the entire research and development system before we can truly prepare and position ourselves ahead of deadly epidemic and pandemic. Only then, can our speed and accuracy in basic discovery be timely translated into clinical and public health needs. The time to act is now.
